# Creating meaningful knowledge exchange between young people and public health practitioners: what role can researchers play?

**DOI:** 10.1177/17579139241230852

**Published:** 2024-08-06

**Authors:** M Barrett, S Shaw, S Jenner, P Hardy-Johnson, S Stanescu, K Woods-Townsend, S Strommer, M Barker

**Affiliations:** MRC Lifecourse Epidemiology Centre, University of Southampton, Southampton General Hospital, Southampton, UK; NIHR Southampton Biomedical Research Centre, University of Southampton and University Hospital Southampton NHS Foundation Trust, Southampton, UK; Centre for Food Policy, City, University of London, London EC1V 0HB, UK; MRC Lifecourse Epidemiology Centre, University of Southampton, Southampton General Hospital, Southampton, UK; NIHR Southampton Biomedical Research Centre, University of Southampton and University Hospital Southampton NHS Foundation Trust, Southampton, UK; MRC Lifecourse Epidemiology Centre, University of Southampton, Southampton General Hospital, Southampton, UK; MRC Lifecourse Epidemiology Centre, University of Southampton, Southampton General Hospital, Southampton, UK; Primary Care, Population Sciences and Medical Education, Faculty of Medicine, University of Southampton, Southampton, UK; Southampton City Council, Southampton, UK; NIHR Southampton Biomedical Research Centre, University of Southampton and University Hospital Southampton NHS Foundation Trust, Southampton, UK; School of Healthcare Enterprise and Innovation, Faculty of Medicine, University of Southampton, Southampton, UK; MRC Lifecourse Epidemiology Centre, University of Southampton, Southampton General Hospital, Southampton, UK; NIHR Southampton Biomedical Research Centre, University of Southampton and University Hospital Southampton NHS Foundation Trust, Southampton, UK; MRC Lifecourse Epidemiology Centre, University of Southampton, Southampton General Hospital, Southampton, UK; NIHR Southampton Biomedical Research Centre, University of Southampton and University Hospital Southampton NHS Foundation Trust, Southampton, UK; School of Health Sciences, Faculty of Environmental and Life Sciences, University of Southampton, Southampton, UK

## Introduction

Many leading global health organisations including the World Health Organization (WHO), the United Nations International Children’s Emergency Fund (UNICEF), the Wellcome Trust, and the Lancet have called for young people to be included in decisions that affect their health and wellbeing.^1–3^ Meaningful engagement with young people is rare. Public consultations are the standard approach used by many statutory bodies to gain insights from the people living in their local communities. The nature of these consultations means that they are often one-off events that do not generate the rapport and trust required to gain meaningful insights. They also do not routinely engage people below the age of 18, partly because of difficulties with securing consent but also because young people have not, until recently, been thought to have views on policy and practice that are worth seeking.^1–3^

**Figure:**
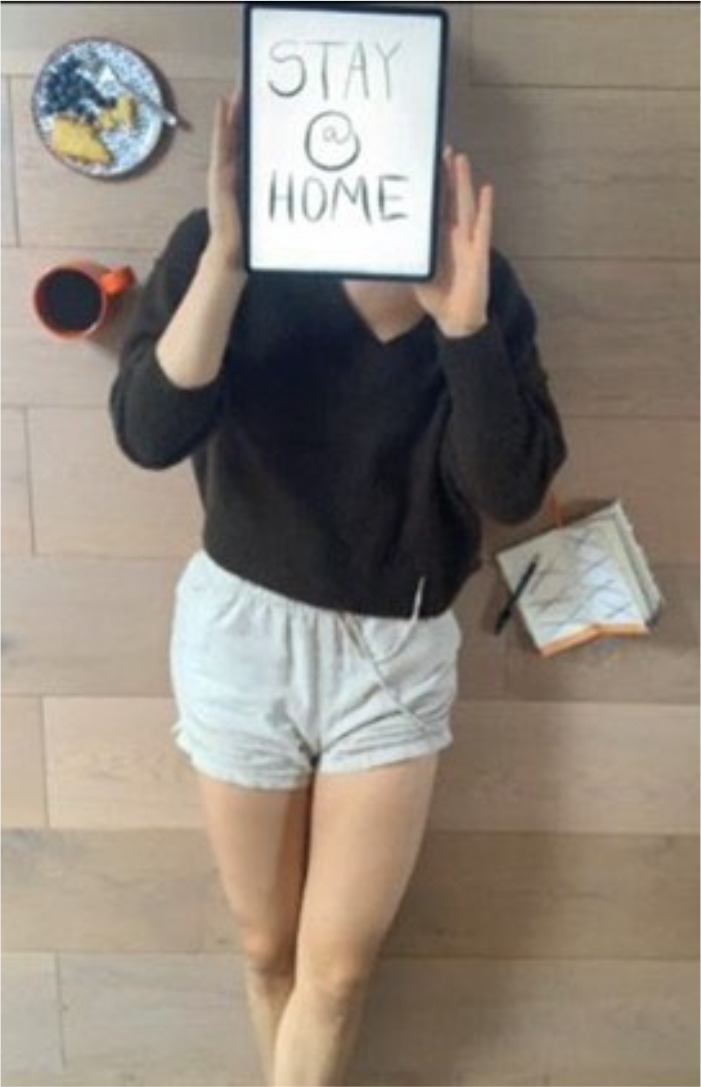


If young people’s views and contributions are to be sought, effective and engaging methods are needed to involve them in meaningful exchange of views and experience. This article presents what we have learned about this process through conducting a longitudinal qualitative research study with young people during the COVID-19 pandemic. We developed methods to facilitate rapid feedback of findings from our discussions with young people to local government organisations to inform their pandemic response. We present this article to share our experience and reflect on how similar approaches could be incorporated into routine research practice to inform decision-making with a speed that is helpful to policy makers.

## Teens and COVID-19 (TeC-19)

The Teens and COVID-19 (TeC-19) study followed 80 adolescents (12–19 years) over the first year of the pandemic (March 2020 to March 2021). Participants formed 10 groups, each taking part in up to seven online focus group discussions (FGDs). Participants were asked about their experiences of lockdown, how they spent their time, their concerns, and their views on the local and national government’s pandemic response. Bespoke semistructured topic guides were developed for each wave of data collection and covered current events at the time. In some FGDs, we used photos and videos of major events, such as protests, to stimulate discussion about pandemic-related issues. During the study period, our research team regularly met online with local government public health teams to share insights from the FGDs to inform their evolving COVID-19 responses.

## Reflections on How Longitudinal Research Methods Facilitated The Dialogue Between Young People and Policy Makers During The COVID-19 Pandemic

### Building trust and rapport

Building strong relationships and rapport with participants is central to qualitative longitudinal research as it helps to develop the trust required for participants to feel comfortable sharing their personal thoughts and feelings.^4^ In TeC-19, we allocated each group a specific research team member who facilitated all of their FGDs throughout the project. This enabled the researchers to build relationships with members of the groups, to revisit topics previously discussed in their groups and to reflect with them on changes over time. This rapport-building approach takes time, which may be a luxury that our public policy colleagues do not have in their work with communities. Our participants were paid £20 for each FGD. We believe that compensating the young people for their participation demonstrated respect for their time, their contribution, and their commitment to the research project.

In this study, the research team acted as conduits for the passage of information between young people and policy makers. As we were not part of the process of policy decision-making and had no responsibility for enforcement, young people were willing to openly and honestly discuss their experiences of and views on adhering to government rules and restrictions. As we had regular updates from the policy makers, we were able to involve the young people in deliberative discussions about the impact of the latest changes in policy and how they thought these might affect themselves and other young people. We chose a deliberative approach to our discussions with young people because they have been shown to be more effective in providing opportunities to discuss trade-offs and expectations involved in changes of policy or practice.^5^ We, and others, have found this to generate more thoughtful and nuanced consideration of issues and therefore to generate more meaningful engagement with both adults and young people alike.^5^

### Collecting high-quality data

TeC-19 was conducted to the rigour and ethical standards expected of academic research. This required careful and consistent documentation of the process of recruitment and data collection as well as keeping a record of pandemic-related events which formed the context for FGDs. The collection and storage of participants’ personal data, particularly for those who are underage, requires clear ethics and safeguarding processes to protect participants’ anonymity and privacy. One of the benefits that academic researchers bring to involving the public in their research is a governance structure that helps ensure all data are collected and managed ethically and safely. The downside of this is that lengthy processes to secure ethics permissions may delay research, making it difficult to deliver information to policy makers fast enough to be useful in making evidence-based decisions. Our ability to broker useful and timely knowledge exchange between young people and policy makers during the early stages of the pandemic was enhanced by the fact that COVID-related studies were prioritised by ethics panels. Methods to prioritise ethics applications may be something that academic institutions should consider to support urgent local government and other stakeholders’ decision-making.

The TeC-19 research team were trained qualitative researchers which enabled them to collect high-quality data. They also had training in asking ‘open-discovery’ questions,^6^ skills in active listening as well as reflecting and summarising participant responses to prompt further in-depth discussion. When collecting feedback from young people about being involved in this research, they told us that these approaches helped them feel their opinions were listened to and valued and that they were making an important contribution to the COVID-19 response.

### Data interpretation to provide meaningful insights

The TeC-19 research team also had an understanding of behaviour change theory and adolescent development. This knowledge allowed us to move beyond simply reporting what young people told us and to reflect on the developmental, social and emotional context of their input, allowing us to provide policy makers with a more meaningful and useful interpretation of the data. Examples include our interpretation of the reasons why young people felt the way they did about mask wearing in schools, weekly testing, the Test, Track and Trace app, and further lockdowns.

We used the principles of rapid qualitative analysis to synthesise the data quickly. Rapid qualitative analysis is particularly suited to time-sensitive studies, and allowed our findings to be shared with policymakers at a speed that reflected the urgency to act in response to the challenges of the pandemic.^7^ Findings from rapid qualitative analyses have been found to be comparable in rigour and validity to more established, time-intensive qualitative methods.^8^ These rapid research methods were frequently applied by researchers during the pandemic when quick decisions had to be made by policy makers on the basis of scant but emerging evidence of the nature of the infection and the likely response of the population.^9,10^

### Reflections and considerations for the future

The COVID-19 pandemic was a unique period in time which presented both opportunities and challenges for researchers to engage meaningfully with young people and policy makers. Due to the tight restrictions placed on young people’s lives and the uncertainty presented by the pandemic, it is possible that they were more motivated to participate in research than they would have been in normal times. They told us that our research discussions gave them something to look forward to and that they enjoyed participating and being financially rewarded for this. In normal times, there are more competing and attractive pressures on their time and attention. It may also be difficult to achieve the same level of commitment from participants when the issues do not have the same immediate impact on their lives as they did during the pandemic. However, conducting the FGDs online and at times designed to suit them made it easy and convenient to join while also facilitating participation from young people in different locations. Alongside fellow researchers, we learnt the value of online data collection for both research teams and participants.

## Conclusion

Using longitudinal research techniques and skills in consultations with young people may facilitate their meaningful engagement with, and involvement in, issues that affect their current and future lives. This involves investing time and resources in building rapport and trust, both of which are essential in allowing young people to share their thoughts and opinions freely and honestly. Skills developed through qualitative research also ensure rigorous and valid conclusions are drawn from what young people report. Moving forward, we have learned and wish to share effective ways to meaningfully engage adolescents in decision-making processes. If young people are genuinely to be architects of their own futures, then we need better mechanisms to allow them to take part and be heard in our public policy debates.

## Notes

Comment from the Editor This article highlights how longitudinal qualitative research can be used to develop relationships and rapport with young people and facilitate meaningful conversations to inform policy and practice decision making. It presents the benefits of researchers working alongside practitioners through a thorough process. It also highlights some challenges often faced in engaging young people and through the timescales of the research process and how these were overcome in this study which took place during the COVID-19 pandemic.
